# Myofibrillar function differs markedly between denervated and dexamethasone-treated rat skeletal muscles: Role of mechanical load

**DOI:** 10.1371/journal.pone.0223551

**Published:** 2019-10-09

**Authors:** Takashi Yamada, Yuki Ashida, Daisuke Tatebayashi, Koichi Himori

**Affiliations:** 1 Graduate School of Health Sciences, Sapporo Medical University, Sapporo, Japan; 2 Research Fellow of Japan Society for the Promotion of Science, Tokyo, Japan; Tohoku University, JAPAN

## Abstract

Although there is good evidence to indicate a major role of intrinsic impairment of the contractile apparatus in muscle weakness seen in several pathophysiological conditions, the factors responsible for control of myofibrillar function are not fully understood. To investigate the role of mechanical load in myofibrillar function, we compared the skinned fiber force between denervated (DEN) and dexamethasone-treated (DEX) rat skeletal muscles with or without neuromuscular electrical stimulation (ES) training. DEN and DEX were induced by cutting the sciatic nerve and daily injection of dexamethasone (5 mg/kg/day) for 7 days, respectively. For ES training, plantarflexor muscles were electrically stimulated to produce four sets of five isometric contractions each day. *In situ* maximum torque was markedly depressed in the DEN muscles compared to the DEX muscles (-74% vs. -10%), whereas there was not much difference in the degree of atrophy in gastrocnemius muscles between DEN and DEX groups (-24% vs. -17%). Similar results were obtained in the skinned fiber preparation, with a greater reduction in maximum Ca^2+^-activated force in the DEN than in the DEX group (-53% vs. -16%). Moreover, there was a parallel decline in myosin heavy chain (MyHC) and actin content per muscle volume in DEN muscles, but not in DEX muscles, which was associated with upregulation of NADPH oxidase (NOX) 2, neuronal nitric oxide synthase (nNOS), and endothelial NOS expression, translocation of nNOS from the membrane to the cytosol, and augmentation of mRNA levels of muscle RING finger protein 1 (MuRF-1) and atrogin-1. Importantly, mechanical load evoked by ES protects against DEN- and DEX-induced myofibrillar dysfunction and these molecular alterations. Our findings provide novel insights regarding the difference in intrinsic contractile properties between DEN and DEX and suggest an important role of mechanical load in preserving myofibrillar function in skeletal muscle.

## Introduction

There is a growing body of evidence showing that loss of muscle strength results not only from muscle atrophy but also from reductions in specific force (i.e. force per cross-sectional area) in a variety of pathophysiological conditions [1, 2]. Denervation (DEN) and glucocorticoids are two major contributors of skeletal muscle atrophy. However, DEN and glucocorticoids appear to have diverse effects on intrinsic contractile properties in rodent skeletal muscles, with a pronounced reduction in specific force in DEN [3] than treatment with dexamethasone (DEX), a synthetic glucocorticoid [4–6]. Although impaired membrane excitability and sarcoplasmic reticulum Ca^2+^ release may contribute to the reduction in specific force, studies using skinned fiber preparations have shown a significant decrease in maximum Ca^2+^-activated specific force (*P*_max_) in all fiber types following DEN [7, 8], suggesting a major role of intrinsic impairment of the contractile apparatus. In contrast, treatment with high-dose DEX results in either reduction [4, 9] or no difference [10] in *P*_max_ in skinned muscle fibers.

One potential explanation for the decrease in *P*_max_ is a reduced number of force-generating cross-bridges, which are formed from the interaction of myosin and actin. Notably, Mozaffar et al. [11] demonstrated that DEN, but not DEX treatment, results in a reduction of myosin heavy chain (MyHC) content. Muscle-specific ring finger 1 (MuRF-1), a member of the tripartite motif family of E3 ubiquitin ligases, has been proposed to target MyHC for polyubiquitination and degradation by the 26S proteasome [12, 13]. Importantly, it has been reported that a depletion of MuRF-1 spares muscle mass in DEN mice [14], but not in DEX mice [13]. Thus, these findings suggest that there are apparently distinct mechanisms between DEN- and DEX-induced muscle atrophy, and that the degradation of muscle proteins other than motor proteins may largely account for the muscle atrophy induced by DEX treatment.

Loss of mechanical stimuli appears to be a dominant factor triggering the proteolysis of myosin and cross-bridge dysfunction in skeletal muscle fibers [15–17]. Although the exact nature of the cellular mechanical sensing is not yet known, neuronal nitric oxide synthase (nNOS), a peripheral member of dystrophin glycoprotein complex, has emerged as a mechanical sensor that affects catabolic gene expression in response to mechanical unloading in skeletal muscle. Investigation on experimental animal models demonstrated that reduced mechanical stress such as unloading or DEN leads to the dislocation of nNOS from sarcolemma to cytoplasm, which results in fiber atrophy by increasing NO production and subsequent activation of forkhead box O (Foxo)/MuRF-1 and atrogin-1 pathway [18]. A deletion of nNOS expression was shown to spare muscle mass induced by unloading or DEN [18].

Reactive oxygen and nitrogen species (ROS/RNS) are increased by a wide variety of conditions that promote muscle weakness. Studies on DEN muscles show activation of NADPH oxidase (NOX) as well as nNOS, which increase superoxide (O_2_^•-^) and NO, respectively [18, 19]. Treatment with DEX increases hydroxyl radicals (OH^•^) production in skeletal muscles, which is formed by metal-catalyzed reaction of O_2_^•-^ and hydrogen peroxide [20]. Intriguingly, recent studies have shown an involvement of ROS/RNS in the translocation of nNOS and subsequent muscle atrophy under unloading conditions [21, 22]. Moreover, ROS/RNS have been shown to directly impair myofibrillar protein function including myosin [23] and actin [24] and hence decrease the force per cross-bridge. ROS/RNS-induced degenerative modifications of cellular proteins accelerate their rate of degradation by calpains [25] and ubiquitin-proteasome pathway [26, 27].

Recently, we have shown reduced *in situ* force production accompanied by decreases in *P*_max_ and MyHC content, and upregulation of ROS/RNS producing enzymes in steroid-denervation (S-D) rats, where animals were exposed to a combination of DEN and DEX treatment [28]. Furthermore, mechanical loading evoked by neuromuscular electrical stimulation (ES) prevented myofibrillar dysfunction and these molecular alterations in S-D rats. However, although these findings suggest a mechanical silencing as a dominant factor triggering the myosin loss and cross-bridge dysfunction in skeletal muscle fibers, mechanistic link between mechanical load and myofibrillar contractility are uncertain due to the involvement of the pharmacological factor. Thus, the subsequent study that compares the effect of ES on myofibrillar function of mechanical-unloaded atrophic condition (i.e., DEN) with that of mechanical-loaded atrophic condition (i.e., DEX) would provide further insight into the role of mechanical load in preserving myofibriller function in skeletal muscle.

In the present study, we tested the following two principle hypotheses: reduction in skinned fiber force was more marked in gastrocnemius (Gas) muscles from DEN than those from DEX and this myofibrillar dysfunction was prevented by mechanical loading evoked by ES; DEN-induced myofibrillar dysfunction involved oxidative stress, nNOS translocation, and degradation of motor proteins, and these changes were also prevented by ES training.

## Materials and methods

### Ethical approval

All experimental procedures were approved by the Committee on Animal Experiments of Sapporo Medical University (No. 16–076). Animal care was in accordance with institutional guidelines.

### Experimental design

To elucidate the role of mechanical stress in myofibrillar function, we assessed the effects of ES training on DEN- and DEX-induced muscle weakness. Male Wistar rats (9 week old, n = 21) were supplied by Sankyo Labo Service (Sapporo, Japan) and were randomly assigned into control (CNT) (n = 6), DEN (n = 9), and DEX (n = 6) groups. Rats were given food and water ad libitum and housed in an environmentally controlled room (24 ± 2°C) with a 12-h light-dark cycle. DEN was induced by removing a 10-mm segment of the sciatic nerve under 2% isoflurane anesthesia. DEX was dissolved in saline at 2 mg/ml and injected intraperitoneally (5 mg/kg) everyday for 7 days. ES training was initiated immediately after DEN or the first DEX injection and was carried out for 7 consecutive days as described previously [28]. Rats were anesthetized by isoflurane inhalation and were placed supine on a platform and their left foot was secured in a foot plate connected to a torque sensor (S-14154, Takei Scientific Instruments) at an angle of 0 degree plantarflexion, with the right legs serving as control. Plantarflexor muscles were stimulated supramaximally (45V) using a pair of surface electrodes that were placed on the skin. Stimulation parameters were set as follows: 0.5 ms monophasic rectangular pulse, 50 Hz, 2 s contraction given every 6 s. Each session consisted of 4 sets of 5 isometric contractions at 5 minutes intervals. Before the final ES session, *in situ* plantar flexor torque (20 and 100 Hz) was measured. Twenty-four hours after the final session, rats were killed by cervical dislocation under isoflurane anesthesia and the Gas muscle was excised from each animal.

### Measurement of Ca^2+^-activated force in skinned fibers

Chemically skinned fibers were prepared as described previously [28]. A part of the excised Gas muscle was pinned out at resting length under paraffin oil and was kept at 4°C. The single muscle fibers were dissected under a stereo-microscope. Four to six skinned fibers were obtained from one whole muscle. A segment of the skinned fiber was connected to a force transducer (Muscle tester, World Precision Instruments) and then incubated with a *N*-2-hydroxyethylpiperazine-*N*’-2-ethanesulfonic acid (HEPES) buffered solution (see below) containing the detergent Triton X-100 (1% (vol/vol), 10 min treatment) to remove all membranous structures. Fiber length was adjusted to optimal length (2.5 μm) by laser diffraction as described previously [29] and the contractile properties were measured at room temperature (24°C).

The solutions used in the skinned fiber analyses were composed of 36 mM Na^+^, 126 mM K^+^, 90 mM HEPES, 8 mM ATP and 10 mM creatine phosphate, and had a pH of 7.09–7.11 at 24°C [30]. The free Mg^2+^ concentration was set at 1.0 mM. The maximum Ca^2+^ solution additionally contained 49.5 mM Ca-EGTA and 0.5 mM free EGTA whereas the relaxation solution contained 50 mM free EGTA. Force-pCa (-log free Ca^2+^ concentration) curves were established with various pCa solutions (pCa 6.4, 6.2, 6.0, 5.8, 5.6, 5.4, and 4.7) prepared by mixing the maximum Ca^2+^ solution and the relaxation solution in appropriate ratios according to the affinity constants reported by Moisescu and Thieleczek [31]. The contractile apparatus was directly activated by exposing the skinned fiber to various pCa solutions and the peak force production in each pCa was measured. The cross-sectional area of fibers was calculated from measurements of their diameters. All skinned fibers were used to determine the maximum Ca^2+^-activated force per cross-sectional area (*P*_max_).

### Determination of MyHC and actin content

Myosin heavy chain (MyHC) and actin content was assessed using quantitative gel electrophoresis and immunoblotting, respectively (immunoblots for actin, see the section “Immunoblots”). To extract whole muscle proteins, muscle pieces were homogenized in ice-cold homogenizing buffer (40 μl/mg wet wt) consisting of: 10 mM Tris maleate; 35 mM NaF; 1 mM NaVO_4_; 1% Triton X-100 (vol/vol); 1 tablet of protease inhibitor cocktail (Roche) per 50 ml. The protein content was determined using Bradford assay [32]. Whole muscle homogenates (2.0 μg total muscle mass) were diluted with SDS-sample buffer: 62.5 mM Tris/HCl; 2% SDS (wt/vol); 10% glycerol (vol/vol); 5% 2-mercaptoethanol (vol/vol); 0.02% bromophenol blue (wt/vol) and were loaded onto 4–15% Criterion TGX Stain Free gels (BioRad) together with known amounts of purified recombinant proteins (rabbit myosin and actin, Sigma), the latter allowing a calibration curve to be generated. Gels were imaged (BioRad Stain Free imager) and the density of MyHC was measured by using Image Lab Software (BioRad). When quantifying absolute amounts of MyHC and actin, the density of the relevant band was converted to an equivalent protein amount, according to the calibration curve derived from the pure protein samples run on the same gel. This amount was expressed relative to the muscle mass.

### Sample preparation

The cytosolic and membrane fraction were extracted from Gas muscle as described previously [33]. Muscle pieces were homogenized in ice-cold homogenizing buffer (10 μl/mg wet wt) consisting of: 250 mM sucrose; 1 mM EGTA, 10 mM HEPES; 10 mM Tris-HCl; 1 tablet of protease inhibitor cocktail (Roche) per 50 ml. Homogenates were centrifuged 10 min at 800 *g* at 4°C, and *supernatant 1* was removed as the soluble fraction. *Supernatant 1* was then centrifuged at 20,000 *g* for 30 min at 4°C. *Supernatant 2* from this spin served as the cytosolic fraction. The remaining pellet was the crude membrane fraction, which was resuspended in buffer containing: 100 mM Tris-HCl; 150 mM NaCl; 5 mM EDTA; 1 mM EGTA; 1% Triton-X 100; 1 tablet of protease inhibitor cocktail (Roche) per 50 ml. The protein content was determined using Bradford assay [32].

### Immunoblots

Immunoblots were performed using: anti-actin (A4700, Sigma), anti-NOX2/gp91^phox^ (ab31092, Abcam), anti-NOX4 (ab133303, Abcam), anti-nNOS (610308, BD Biosciences), anti-endotherial NOS (eNOS) (610296, BD Biosciences). Aliquots of the whole muscle proteins, cytosolic proteins, or membrane proteins were diluted with SDS-sample buffer. Proteins were separated on 4–15% Criterion TGX Stain Free gels (BioRad). Then proteins were transferred onto polyvinylidine fluoride membranes. Membranes were blocked in 3% (wt/vol) non-fat milk, Tris-buffered saline containing 0.05% (vol/vol) Tween 20, followed by incubation with primary antibody overnight at 4°C. Membranes were then washed and incubated for 1 h at room temperature (~24°C) with secondary antibody (1:10,000, donkey-anti-rabbit or donkey-anti-mouse, BioRad). Images of membrane were collected following exposure to chemiluminescence substrate (Millipore) using a charge-coupled device camera attached to ChemiDOC MP (BioRad), and Image Lab Software was used for detection as well as densitometry.

### Quantitative real-time PCR

Real-time PCR was used to quantify the mRNA levels for atrogin-1 and MuRF-1 in frozen Gas muscle tissue as described previously [28]. Briefly, total RNA was extracted with TORIZOL reagent (Invitrogen, Carlsbad, CA), and the purity and yield of the total RNA extracted was determined by absorbance of aliquots at 260 and 280 nm (Thermo Scientific Nanodrop Light). Total RNA was reverse-transcribed to cDNA using Prime Script RT Reagent Kit (Takara). Synthesized cDNA was then amplified on the Applied Biosystems 7500 with Premix Ex Taq^TM^ kit (Takara). The following Taqman Probes (Applied Biosciences^TM^) were used: rat atrogin-1 (Ebxo32, Rn00591730_m1), rat muscle RING-Finger protein 1 (MuRF-1) (Trim63, Rn00590197_m1), rat GAPDH (Rn01775763_g1). All samples were run in duplicate. Relative amounts of target mRNA was determined using the comparative threshold cycle method (ΔΔCT). Expression of target genes was normalized to the corresponding expression level of GAPDH.

### Autolysis of calpain-1

Muscle pieces of approximately 100 mg were diluted in nine volumes (mass/vol) of ice-cold homogenizing buffer: 5 mM EDTA; 5 mM EGTA; 20 mM Tris; 0.001% (mass/vol) pepstatin A; 0.001% (mass/vol) 4-(2-aminoethyl)-benzenesulfonyfluoride (AEBF); 1 mM dithiothreitol (DTT); 0.5 mM phenylmethylsulfonylfluoride (pH 7.4) and homogenized on ice using a hand-held glass homogenizer. Muscle proteins (20 μg/lane) were separated on a 7% SDS-polyacrylamide gel and immunoblotting was performed using anti-calpain-1 antibody (C0355, Sigma) as described previously [28, 34]. The amount of total calpain-1 was quantified by calculating the sum of the density of all the bands (i.e., autolyzed and unautolyzed) and the amount of autolyzed calpain-1 was expressed as a percentage of total calpain-1 in the same muscle sample.

### Statistics

Data are presented as mean ± SEM. Statistical significance of the difference between the groups was determined with a two-way ANOVA followed by the Tukey test for multiple comparisons (SigmaPlot 13, Systat Software, Inc.). A *P* value less than 0.05 was regarded as statistically significant.

## Results

### Body and muscle weights

The body weight of DEX, but not DEN, rats was significantly lower than that of the CNT group (*P* < 0.05) (Table 1). Absolute weight for the Gas muscle was decreased by 24% and 17% in DEN and DEX rats, respectively (*P* < 0.05) (Table 1). The Gas muscle weight was higher in the DEN+ES group than in the DEN group (*P* < 0.05). In contrast, there was no difference in the Gas muscle weight between DEX and DEX+ES group.

**Table 1:** Body and muscle weight of control, denervated, dexamethasone-treated rats.

	CNT	CNT+ES	DEN	DEN+ES	DEX	DEX+ES
n	6	6	9	9	6	6
Body (g)	237 ± 2		225 ± 3		192 ± 4^a^	
Gas (mg)	1125 ± 23	1179 ± 23	851 ± 19^a^	923 ± 19^b^^,^^c^	938 ± 23^a^^,^^b^	986 ± 23^c^

Values are means ± SEM. CNT, control; DEN, denervation; DEX, dexamethasone; ES, neuromuscular electrical stimulation; n, number of samples; Gas, gastrocnemius muscle.

^a^*P*<0.05 *vs*. CNT

^b^*P* < 0.05 *vs*. DEN, and

^c^*P*<0.05 *vs*. CNT+ES.

### Myofibrillar dysfunction was more marked in DEN than DEX rats

**Fig 1:**
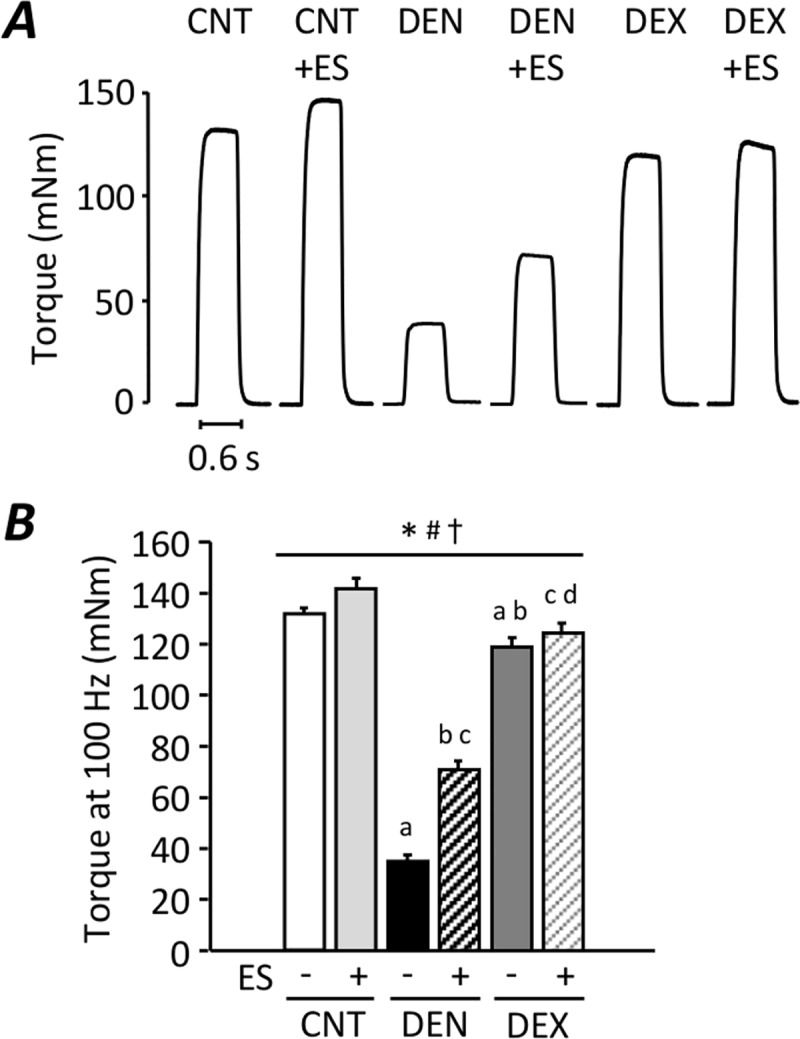
*In situ* torque depression was more marked in DEN than in DEX rats. Representative original records of 100 Hz tetanic torque in plantarflexor muscles from control (CNT), denervation (DEN), and dexamethasone (DEX) rats with or without neuromuscular electrical stimulation (ES) training **(A)**. Absolute torque at 100 Hz stimulation frequencies **(B)**. Bars show the mean and SEM results from 6–9 muscles per group. Statistical significance was set at *P*<0.05: main effect of *treatment (i.e., DEN and DEX) and ^#^ES; ^†^interaction of treatment and ES; difference versus ^a^CNT, ^b^DEN, ^c^CNT+ES, and ^d^DEN+ES.

At the 20 Hz stimulation frequency, there was no difference in *in situ* plantarflexor torque between the groups. In contrast, compared to the CNT muscles (132 ± 4 mNm), DEN (35 ± 3 mNm) and DEX (119 ± 4 mNm) induced a depression in *in situ* maximum torque at the 100 Hz stimulation freqeuncy (*P* < 0.05) (Fig 1A & 1B). Notably, the reduction in maximum torque in the DEX group (-10%) was less pronounced than in the DEN group (-74%) (*P* < 0.05). ES training ameliorated the maximum torque depression in DEN, but not DEX, group (*P* < 0.05).

**Fig 2:**
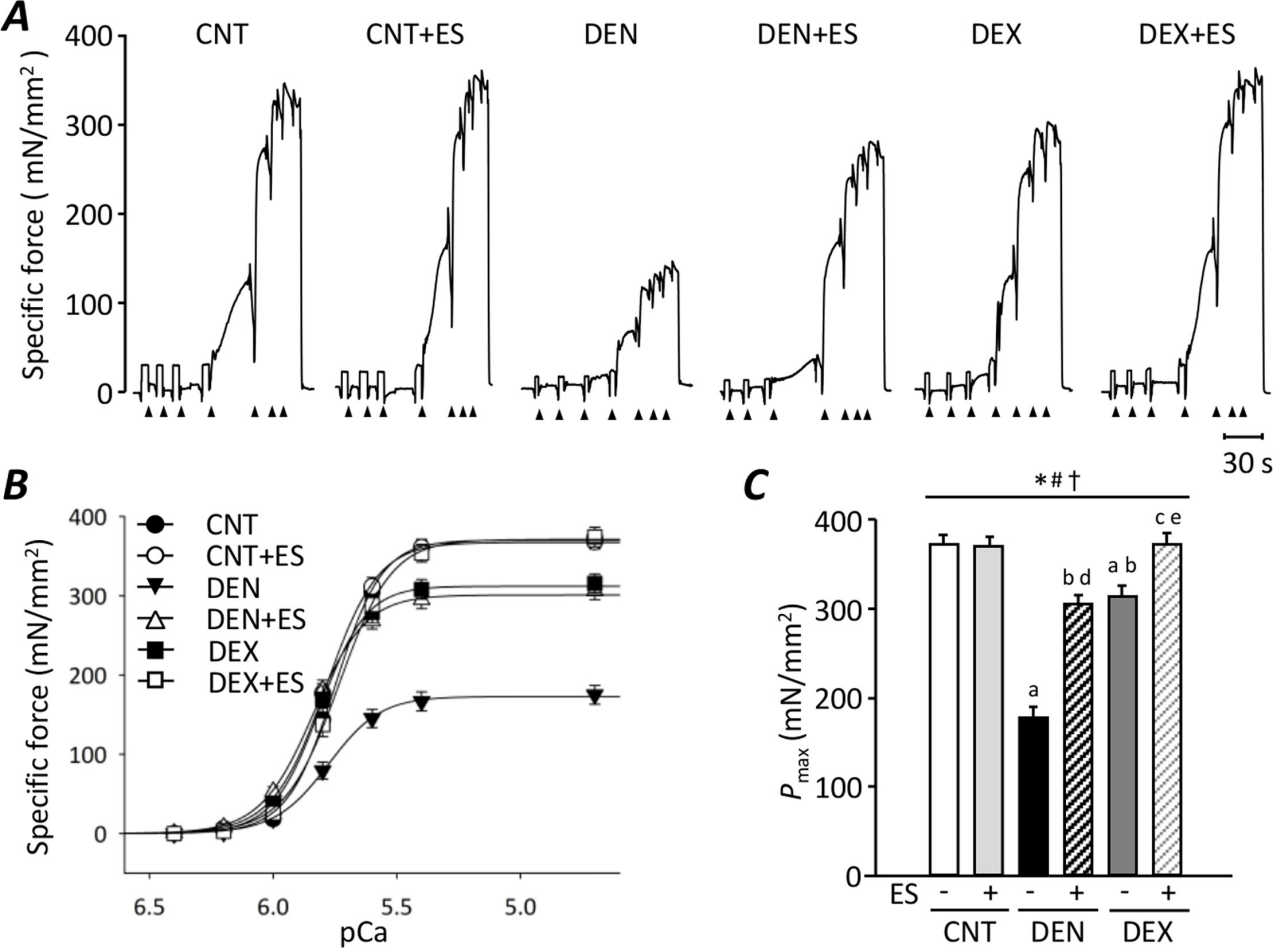
Myofibrillar dysfunction was more marked in DEN than in DEX rats. Representative original records of Ca^2+^-activated force in chemically skinned fibers from gastrocnemius muscles of control (CNT), denervation (DEN), and dexamethasone (DEX) rats with or without neuromuscular electrical stimulation (ES) training **(A)**. Fibers were exposed to solutions at progressively higher free Ca^2+^ concentration: pCa 6.4, 6.2, 6.0, 5.8, 5.6, 5.4, and 4.7. Specific force-frequency relationship **(B)** and the maximum Ca^2+^-activated force (*P*_max_) **(C)**. Data presented as mean and SEM from 20–25 fibers per group. Statistical significance was set at *P*<0.05: main effect of *treatment (i.e., DEN and DEX) and ^#^ES; ^†^interaction of treatment and ES; difference versus ^a^CNT, ^b^DEN, ^c^ DEX, ^d^CNT+ES, and ^e^DEN+ES.

Fig 2A shows the typical traces of Ca^2+^-activated force in skinned fibers from CNT, DEN, and DEX group with or without ES training. Force-pCa curves constructed from mean data show that *P*_max_ was reduced in fibers from DEN (175 ± 12 mN/mm^2^) and DEX (311 ± 11 mN/mm^2^) muscles relative to those of CNT muscles (369 ± 11 mN/mm^2^) (*P* < 0.05) (Fig 2B & 2C). The reduction in *P*_max_ was more marked in fibers from DEN (-53%) than DEX (-16%) muscles (*P* < 0.05). Importantly, ES training almost completely prevented the DEN- and DEX-induced reduction in *P*_max_ (*P* < 0.05).

### Myosin and actin content were decreased in DEN muscles, but not in DEX muscles

**Fig 3:**
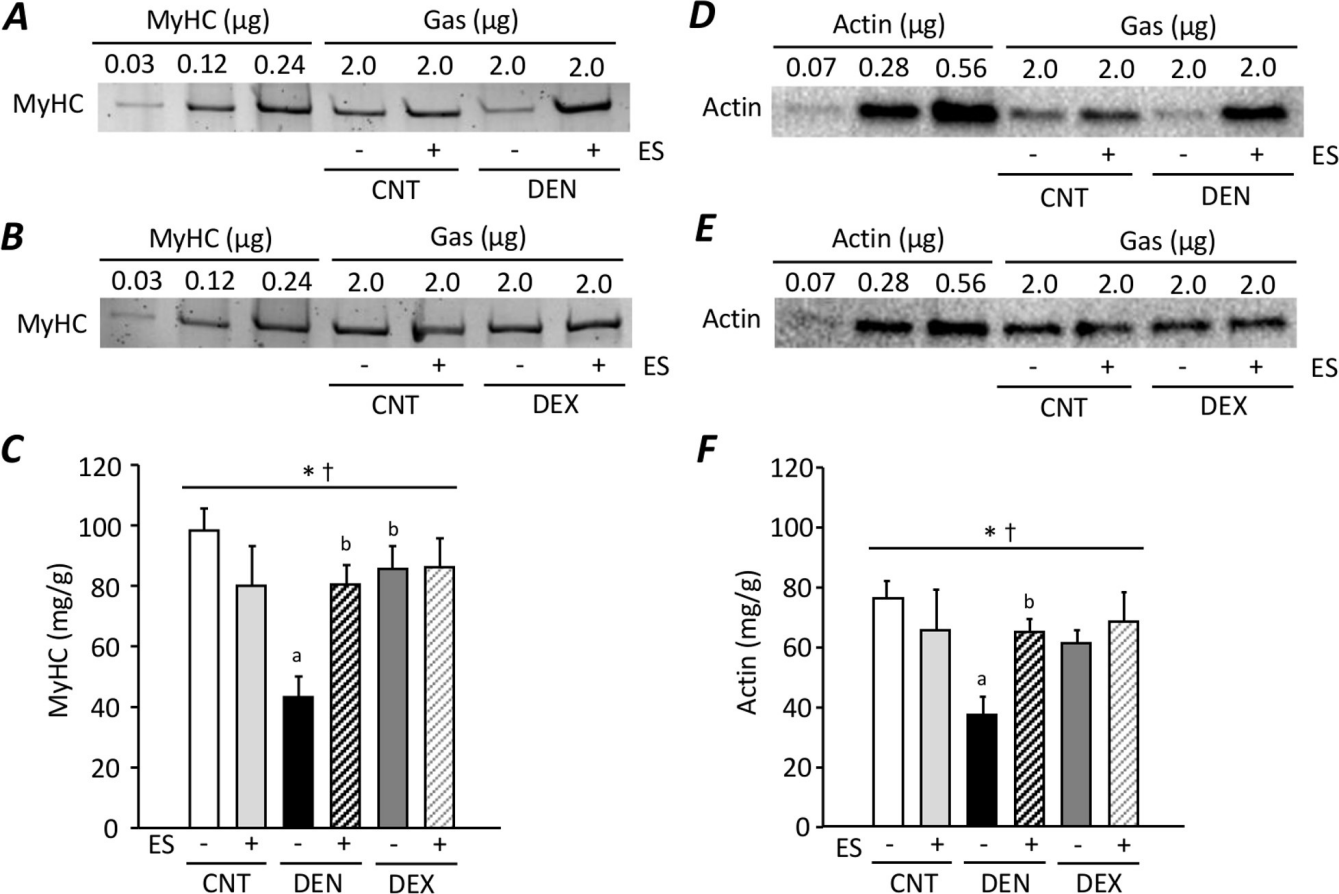
MyHC and actin content were decreased in DEN muscles, but not in DEX muscles. Purified recombinant myosin heavy chain (MyHC) (0.03–0.24 μg), actin (0.07–0.56 μg), and total muscle homogenates (2 ug total muscle mass) from the gastrocnemius (Gas) muscles from control (CNT), denervation (DEN), and dexamethasone (DEN) rats with or without neuromuscular electrical stimulation (ES) training were run on the same gel. Representative stain free images of MyHC (**A, B**) and immunoblots for actin (**D, E**). MyHC (**C**) and actin (**F**) content of the Gas muscle homogenates were calculated using calibration curves, which were obtained by plotting band density of known amount of purified MyHC and actin in each membrane. Bars show the mean and SEM results from 6–8 muscles per group. Statistical significance was set at *P*<0.05: main effect of *treatment (i.e., DEN and DEX); ^†^interaction of treatment and ES; difference versus ^a^CNT and ^b^DEN.

To assess the absolute amount of MyHC and actin following DEN and DEX, small amount of unfractionated Gas muscle homogenate (2 μg) were run with known amounts of purified MyHC (0.03–0.24 μg) and actin (0.07–0.56 μg) (Fig 3A, 3B, 3D and 3E), which allowing a calibration curve to be generated. We observed 56% and 51% decrease in MyHC and actin content in DEN muscles, respectively, which were fully prevented by ES training (*P* < 0.05) (Fig 3C & 3F). Conversely, compared to the CNT muscles, there were no changes in MyHC and actin content in DEX muscles.

### ES training prevents the upregulation of ROS/RNS producing proteins in DEN muscles

**Fig 4:**
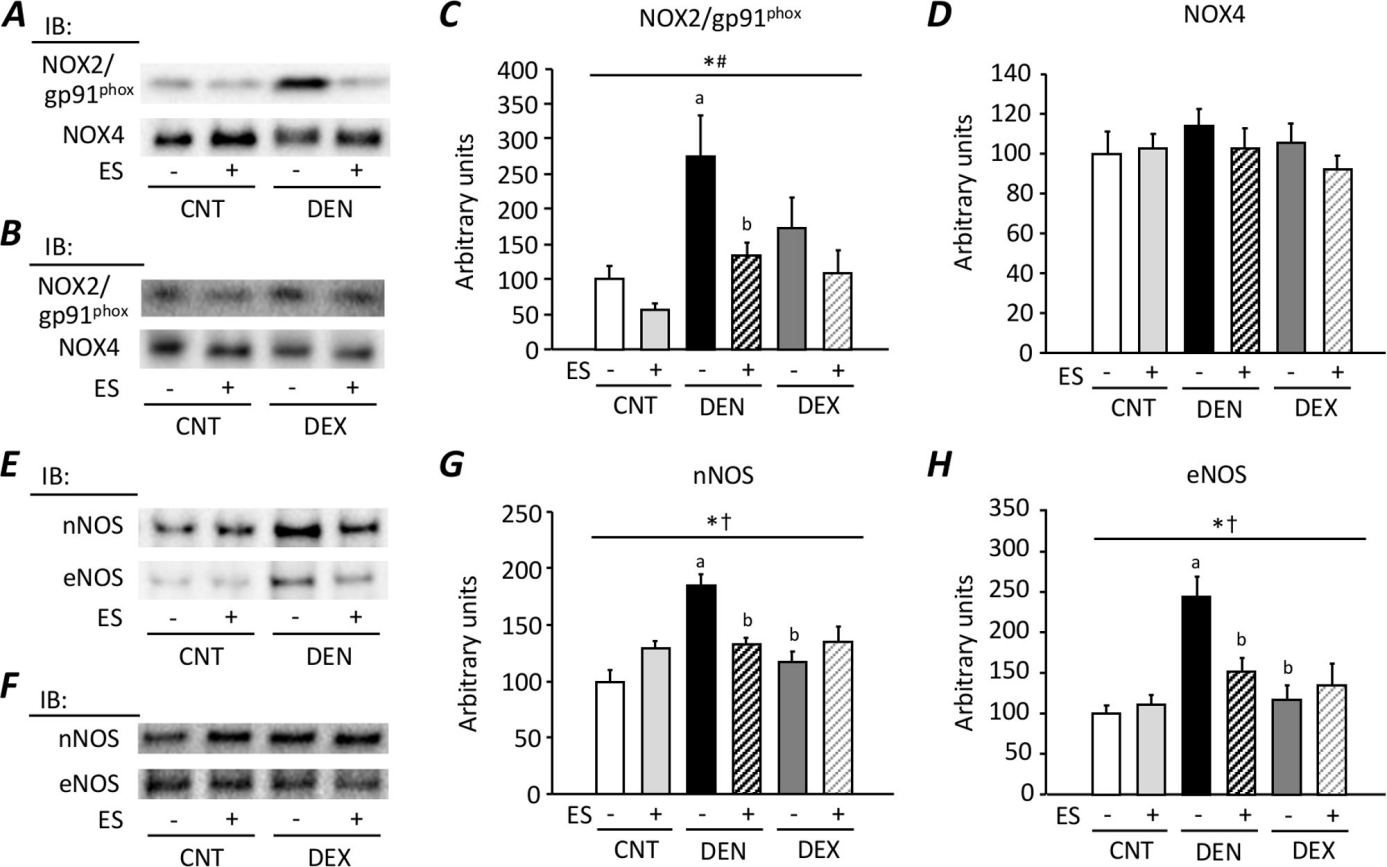
Neuromuscular electrical stimulation (ES) training prevents the upregulation of ROS/RNS producing proteins in DEN muscles. Representative immunoblots illustrating the levels of NADPH oxidase (NOX2/gp91^phox^), NOX4 (**A, B**), neuronal nitric oxide synthase (nNOS), and endotherial NOS (eNOS) (**E, F**) in whole muscle protein of gastrocnemius muscles from control (CNT), denervation (DEN), and dexamethasone (DEX) rats with or without ES training. The levels of NOX2/gp91^phox^ (**C**), NOX4 (**D**), nNOS (**G**), and eNOS (**H**) expression were normalized to the total protein content seen in the stain free images. Bars show the mean and SEM results from 5–9 muscles per group. Statistical significance was set at *P*<0.05: main effect of *treatment (i.e., DEN and DEX) and ^#^ES; ^†^interaction of treatment and ES; difference versus ^a^CNT and ^b^DEN.

The expressions levels of NOX2/gp91^phox^, nNOS, and eNOS, but not NOX4, were significantly increased in DEN muscles (*P* < 0.05) (Fig 4A–4H). ES training prevented the upregulation of these enzymes in DEN muscles (*P* < 0.05). In contrast, DEX treatment did not affect the expression levels of NOXs and NOSs.

### ES training reduces dislocation of nNOS from the sarcolemma and limits the increase in mRNA expression of ubiquitin ligases in DEN muscles

**Fig 5:**
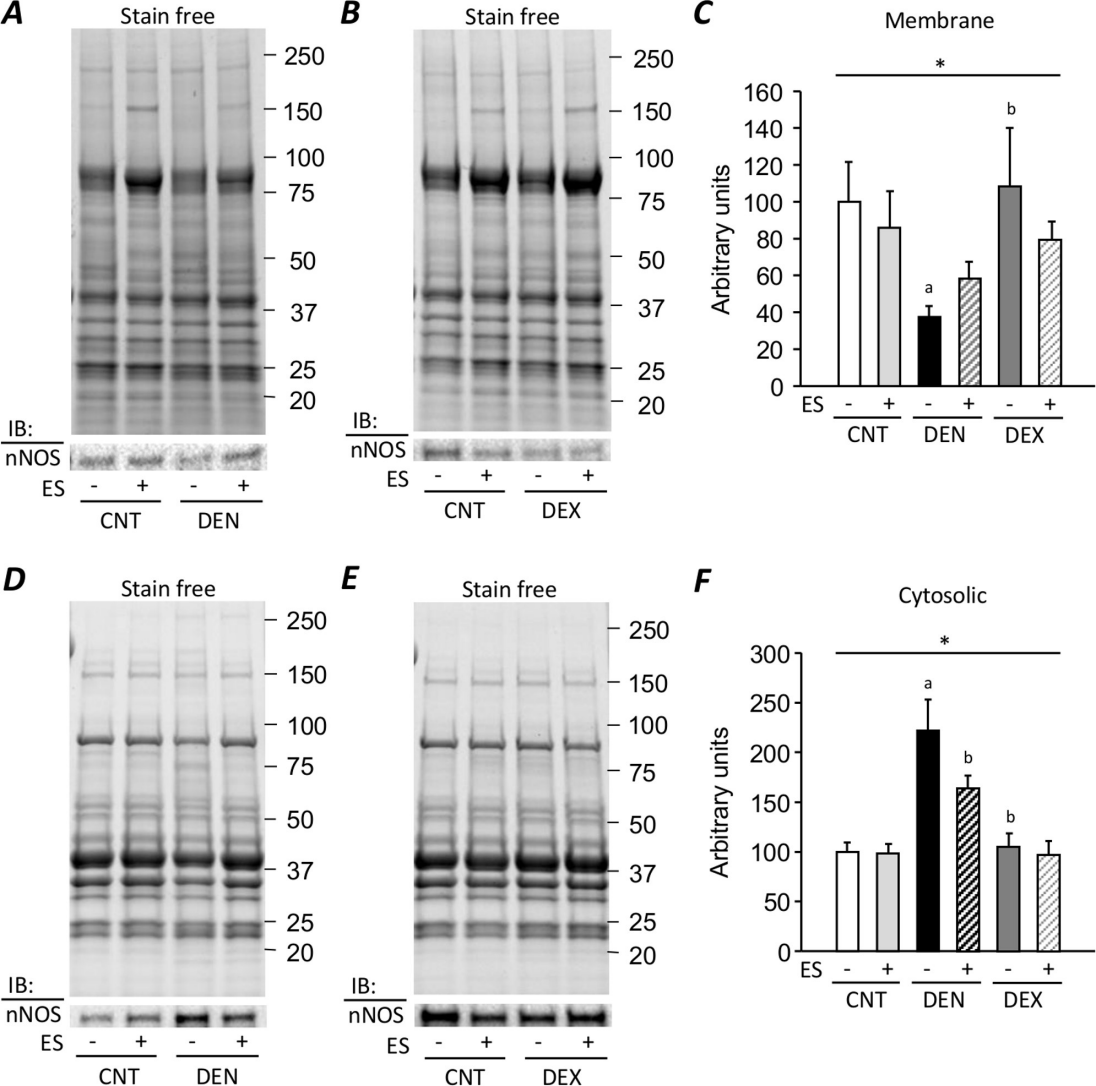
Neuromuscular electrical stimulation (ES) training reduces dislocation of nNOS from the sarcolemma in DEN muscles. Representative western blots illustrating the nNOS in the membrane (**A, B**) and the cytosolic (**D, E**) fraction of gastrocnemius muscles from control (CNT), denervation (DEN), and dexamethasone (DEX) rats with or without ES training. The expression level of nNOS was normalized by the total protein content seen in the stain free images (**C, F**). Bars show the mean and SEM results from 5–9 muscles per group. Statistical significance was set at *P*<0.05: main effect of *treatment (i.e., DEN and DEX); difference versus ^a^CNT and ^b^DEN.

In DEN muscles, the expression level of nNOS was significantly decreased in the membrane fraction (Fig 5A–5C), whereas it was markedly increased in the cytosolic fraction (P < 0.05) (Fig 5D–5F).

**Fig 6:**
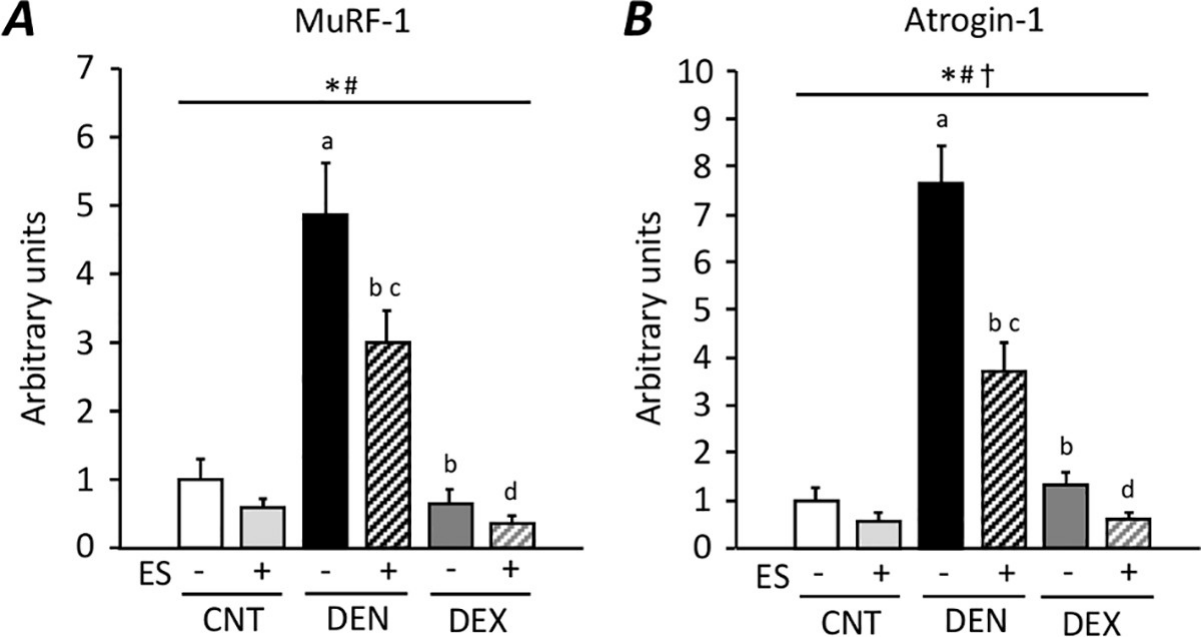
Neuromuscular electrical stimulation (ES) training limits the increase in mRNA expression of ubiquitin ligases in DEN muscles. The expression levels of MuRF-1 (**A**) and atrogin-1 (**B**) mRNA were normalized to the glyceraldehyde-3-phosphate dehydrogenase mRNA and expressed as fold change of the mean CNT value, which was set to 1. Bars show the mean and SEM results from 6–9 muscles per group. Statistical significance was set at *P*<0.05: main effect of *treatment (i.e., DEN and DEX) and ^#^ES; ^†^interaction of treatment and ES; difference versus ^a^CNT, ^b^DEN, ^c^CNT+ES, and ^d^DEN+ES.

Moreover, compared to the CNT group, DEN induced a 4.9-fold and 7.7-fold increase in the mRNA expression of the muscle-specific ubiquitin ligases MuRF-1 and atrogin-1, respectively (P < 0.05) (Fig 6A & 6B). ES training suppressed the dislocation of nNOS and the increased expression levels of MuRF-1 and atrogin-1 mRNA in DEN muscles (P < 0.05). On the other hand, there were no changes in the distribution of nNOS and the expression levels of MuRF-1 and atrogin-1 mRNA in DEX muscles.

### ES training prevents the activation of calpain-1 in DEN muscles

**Fig 7:**
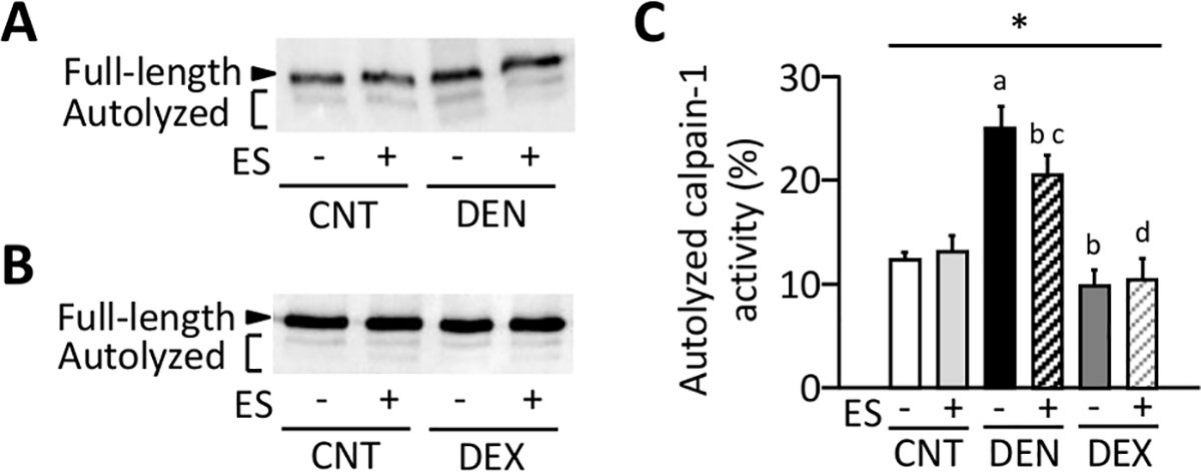
Neuromuscular electrical stimulation (ES) training prevents the activation of calpain-1 in DEN muscles. Representative western blots illustrating the autolysis of calpain-1 of gastrocnemius muscles in control (CNT), denervation (DEN), dexamethasone (DEX) rats with or without ES training (**A, B**). The content of autolyzed calpain-1 was expressed as a percentage of the total calpain-1 in the same muscle sample (**C**). Bars show the mean and SEM results from 5–9 muscles per group. Statistical significance was set at *P*<0.05: main effect of *treatment (i.e., DEN and DEX); difference versus ^a^CNT, ^b^DEN, ^c^CNT+ES, and ^d^DEN+ES.

Ca^2+^ triggers an autolytic process in calpain-1 and reduces the [Ca^2+^] required for its activation from 400–800 to 50–150 μM [35]. Full-length calpain-1 exists as an 80-kDa protein and can be autolyzed to proteins of 78- and 76-kDa. Immunoblot analysis showed that the amounts of autolyzed calpain-1 were elevated in DEN muscles and this was decreased by ES training (*P* < 0.05) (Fig 7A & 7C). In contrast, there was no difference in the amounts of autolyzed calpain-1 between CNT and DEX group.

## Discussion

In the present study, the magnitude of *in situ* maximum torque depression was larger than that of muscle atrophy in DEN (-74% vs. -24%, respectively), but not DEX muscles (-10% vs. -17%, respectively). These results are consistent with the previous studies showing that the reduction in specific force is much more marked in DEN than in DEX treatment [3–6]. In order to clarify the mechanism underlying these differences, we assessed the myofibrillar function in skinned fibers and found that the magnitude of reduction in *P*_max_ was obviously higher in DEN than in DEX group (-53% vs. -16%). Thus, although DEN and DEX are potent inducer of skeletal muscle atrophy, DEN is more likely to develop myofibrillar dysfunction than DEX treatment, which accounts for impaired *in situ* torque production in DEN muscles. Importantly, we provide the first evidence that this myofibrillar dysfunction was almost completely prevented by ES training, suggesting the mechanical load as an important factor in preserving myofibrillar function.

Larsson and colleagues [16, 36] have demonstrated that passive mechanical load alleviates the marked decline in specific force and myosin loss in mechanically ventilated, deeply sedated, and pharmacologically paralyzed rats, where mechanical stimuli was completely absent. Moreover, they demonstrated that the improved maintenance of muscle mass and function can be a consequence of a reduced oxidative stress [36]. Similar results were obtained in the present study that mechanical load evoked by ES fully prevents reduction in *P*_max_ and MyHC as well as an increase in the ROS/RNS generating enzymes in DEN muscles. Interestingly, exercise training has been shown to decrease NOX2 activity and restore skeletal muscle mass and function in several pathological conditions [37, 38]. Thus, these findings suggest that mechanical silencing causes the increased ROS/RNS production and that preserved myofibrillar function resulted from ES training may reflect inhibition of oxidative stress in DEN muscles (see below).

In principle, the decrease in *P*_max_ in skinned fiber can be due to a decreased number of force producing cross-bridges and/or impaired cross-bridge function with decreased force per cross-bridge. Although precise mechanism behind the DEN-induced myofibrillar dysfunction is not well understood [7], loss of motor proteins has been shown to be associated with reduced skinned fiber force in a variety of pathophysiological conditions [39–44]. Moreover, actin-activated ATPase activity in isolated myosin heads has been shown to be inhibited by ROS/RNS, which reduces maximum force [23]. We recently showed a significant reduction in skinned fiber force in S-D rats, where loss of MyHC content and upregulation of ROS/RNS producing enzymes are concomitantly induced [28]. Present study demonstrated that DEN, but not DEX, elicites the reduction in MyHC and actin content, which was associated with upregulation of NOX2/gp91^phox^, a major source of O_2_^•-^ in skeletal muscle. Therefore, DEN may result in severe myofibrillar dysfunction due to the combined effect of a decreased number of cross-bridges and depression in force per cross-bridge induced by ROS/RNS.

Intracellular protein degradation is thought to be a precisely controlled and highly specific process [45]. In agreement with the previous studies [11, 45], the increase in catabolism of myofibrillar proteins was especially elevated in DEN muscles compared to DEX muscles. Increased rates of protein degradation are mainly induced by the activation of ubiquitin proteasome system in unloaded muscles [14]. Two E3 ubiquitin ligases, MuRF1 and atrogin-1, are upregulated in several skeletal muscle atrophy models. Although MuRF-1 has been proposed to target MyHC and other thick filament proteins for polyubiquitination and degradation by the 26S proteasome in DEN and DEX muscles, MuRF-1 deletion spares muscle atrophy in DEN, but not DEX, muscles[12–14]. Moreover, Furlow et al. [46] showed that the lack of MuRF-1 causes fewer changes in the gene expression patterns of contractile proteins in the DEX-induced atrophy than DEN-induced atrophy. In line with these findings, expression levels of MuRF-1 mRNA were significantly elevated in DEN muscles compared to DEX muscles. Thus, catabolism of cellular fractions appears to be independently regulated and myofibrillar proteins seem selectively degraded in DEN muscles, where mechanical load is nearly absent.

nNOS, a peripheral member of dystrophin glycoprotein complex, is proposed as a mechanical sensor in skeletal muscle. Suzuki et al. [18] have demonstrated that dislocation of nNOS leads to the production of NO and activates the ubiquitin-proteasome pathway in unloading- and DEN-induced muscle atrophy. Consistent with this, we observed that DEN, but not DEX, results in translocation of nNOS from the membrane to the cytoplasm and increased expression levels of MuRF-1 and atrogin-1 mRNA, which was accompanied by loss of motor proteins MyHC and actin. Remarkably, we also showed that mechanical loading evoked by ES inhibits DEN-induced muscle atrophy and these molecular alterations. Previous study on human vastus lateralis muscles demonstrated that resistance exercise prevents reduced sarcolemmal nNOS immunostaining following 12 weeks of bed rest [47]. Thus, these data suggest that sarcolemmal nNOS plays an important role in preserving myofibrillar function in skeletal muscles. On the contrary, in DEX treated animals, mechanical loading is kept intact and hence nNOS is located in the membrane, which in turn would limit the degradation of myofibrillar proteins induced by activation of ubiquitin-proteasome pathway.

Although the mechanism underlying translocation of nNOS are not well understood, increase in ROS/RNS can be involved in this process. It has been shown that EUK-134, a cell-permeable mimetic of SOD and catalase, abolishes dislocation of nNOS induced by mechanical unloading [21]. Moreover, loss of sarcolemmal nNOS activity was associated with increased oxidative stress in unloaded rat skeletal muscle [22]. In line with this, we observed concomitant increases in NOX2/gp91^phox^ expression and translocation of nNOS in DEN muscles, which were normalized by ES training. Therefore, these data suggest that nNOS translocation and subsequent catabolic signaling are modulated by redox signaling that is, at least partially, attributed to the absence of mechanical loading. On the other hand, increased ROS/RNS production has been linked to the calpain-mediated myofibrillar protein degradation [25]. Our results show an increased levels of autolyzed active calpain-1 in DEN muscles and this was prevented by ES training. Thus, the degradation of motor proteins in DEN was likely to be mediated by activation on ubiquitin proteasome pathway and calpain-1 due to increased ROS production by NOX2/gp91^phox^.

In addtition to the post-translational degradation, DEN-induced loss of MyHC and actin can be due to pre-translational defects. It has been demonstrated in rat skeletal muscle that DEN, but not DEX, treatment significantly reduces the expression levels of MyHC and actin mRNA, despite an paradoxical increase in total RNA concentration [11]. Several studies have shown an increased protein synthesis and an activation of the mammalian target of rapamycin (mTOR) signaling, a critical regulator of protein translation and cellular growth, following DEN [48, 49]. In contrast, glucocorticoid was shown to inhibit protein synthesis mainly due to the inhibition of mTOR [50], which would account for muscle wasting seen in DEX muscles. Thus, taking these data and our findings into account, it appears that mechanical silencing selectively suppresses the myofibrillar protein synthesis, whereas glucocoriticoid induces the reduced protein synthesis other than myofibrillar proteins in skelatal muscle.

ES training improved the force development in skinned fiber of DEX-treated muscles while *in situ* torque remained low compared with the CNT group (see Figs 1 & 2). Similar phenomenon was also observed in the DEN group, in which the torque production was significantly increased by ES training but the magnitude of the increase seems more marked in skinned fibers. We have previously demonstrated that ES training, which is exactly the same as the present study, spares the myofibrillar dysfunction, but has little effect on the activation failure (i.e., membrane hypoexcitability) in skelatal muscles from S-D rat [28]. Thus, it is most likely that ES training used in our study does not protect against activation impairment induced by DEN and DEX and hence is less effective in depolarization-induced torque as compared to Ca^2+^-activated myofibrillar force production.

## Conclusions

We here show that DEN can cause severe myofibrillar dysfunction compared to DEX. This dysfunction is likely to be mediated by increased production of ROS/RNS and subsequent translocation of nNOS, which result in loss of motor proteins due to the activation of ubiquitin proteasome pathway and calpain-1. ES training abolished these deleterious alterations and preserved myofibrillar function. Thus, our data suggest that mechanical silencing involves in triggering a major part of myofibrillar dysfunction, and this was potently counteracted by ES-induced mechanical loading.

## Supporting information

S1 Fig Original uncropped blots used for Figs 3, 4, 5 and 7. Image was captured using a charge-coupled device camera attached to ChemiDOC MP (BioRad) and Image Lab Software was used for detection as well as densitometry. The part of the blot or gel shown in the manuscript is within the red box.
(PDF)
