# Supplementary material for: Myofibrillar function differs markedly between denervated and dexamethasone-treated rat skeletal muscles: Role of mechanical load
Source: PLoS One. 2019 Oct 9;14(10):e0223551. doi: 10.1371/journal.pone.0223551 (PMC6785062; doi:10.1371/journal.pone.0223551)
Supplement: S1 Fig — Image was captured using a charge-coupled device camera attached to ChemiDOC MP (BioRad) and Image Lab Software was used for detection as well as densitometry. The part of the blot or gel shown in the manuscript is within the red box. (PDF) [file pone.0223551.s001.pdf]

Fig. 3A

Stain free images

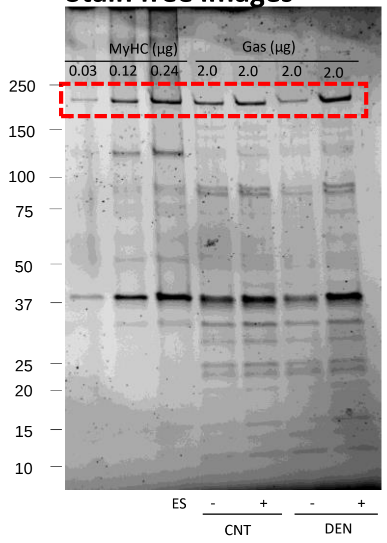

Fig. 3D

IB: actin

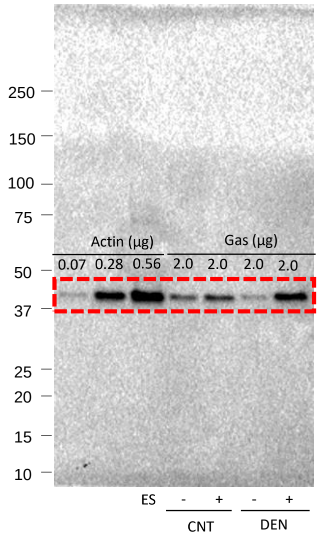

Fig. 3B

Stain free images

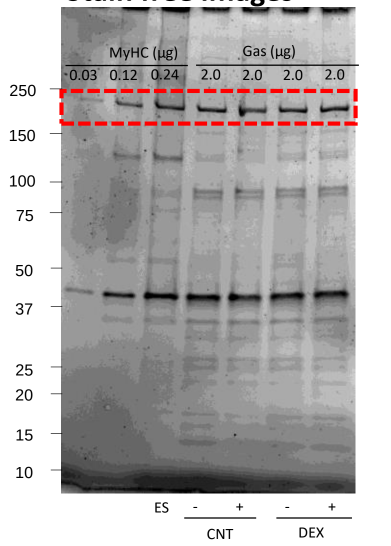

Fig. 3E

IB: actin

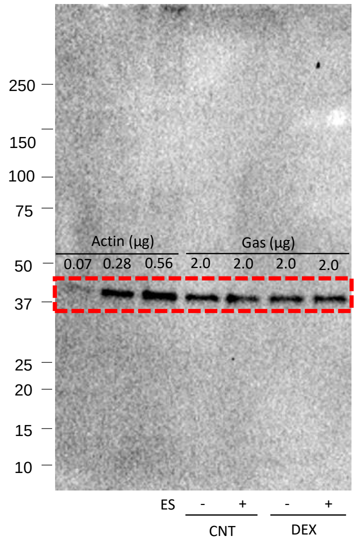

S1_raw_images. Original uncropped blots used for Fig. 3. Image was captured using a charge-coupled device camera attached to ChemiDOC MP (BioRad) and Image Lab Software was used for detection as well as densitometry. The part of the blot or gel shown in the manuscript is within the red box.

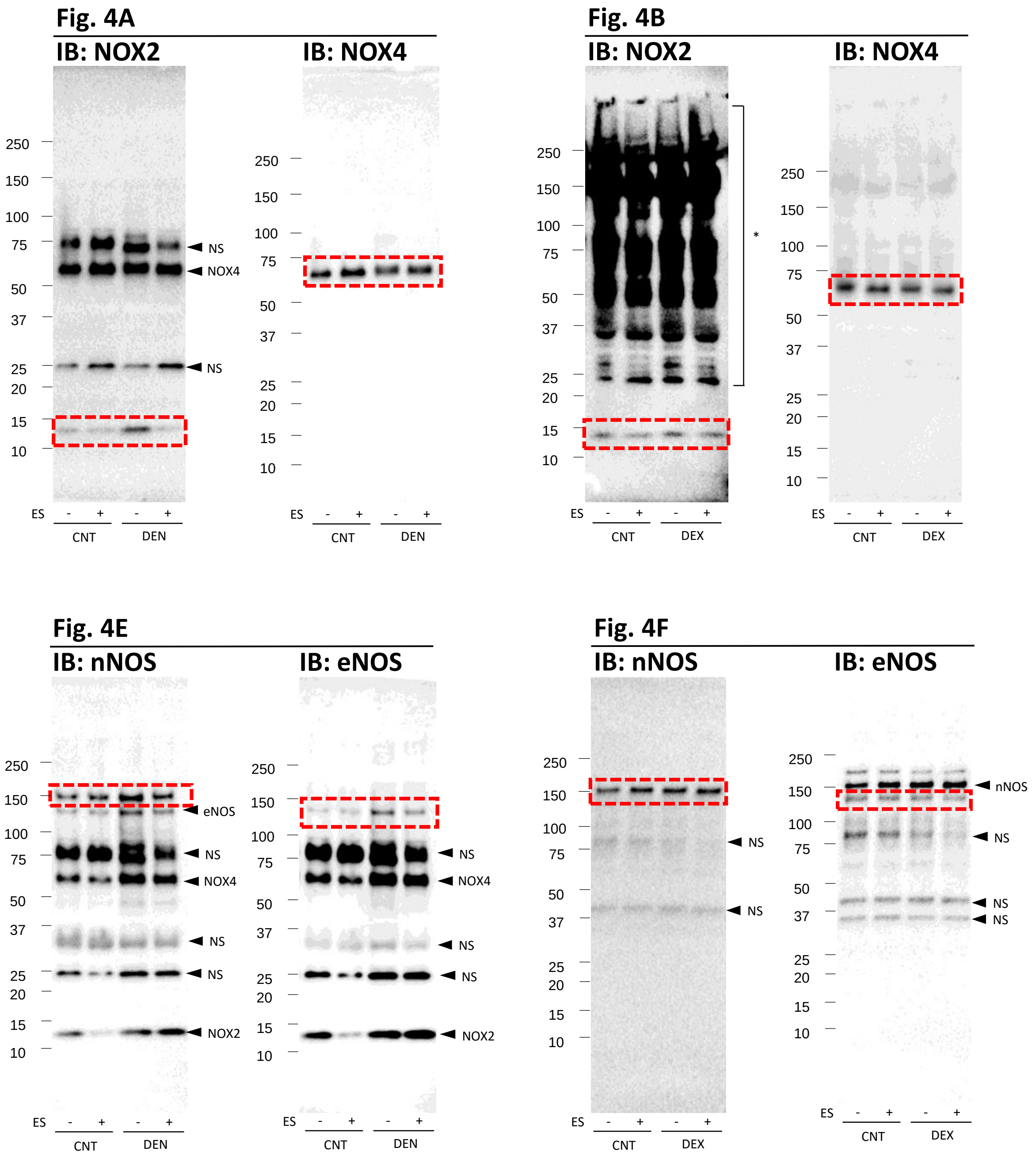

S1_raw_images. Original uncropped blots used for Fig. 4. Image was captured using a charge-coupled device camera attached to ChemiDOC MP (BioRad) and Image Lab Software was used for detection as well as densitometry. The part of the blot shown in the manuscript is within the red box.
NS: non-specific binding. *Blots for p-p70S6K, p-rpS6, total AKT, and their NSs.

Fig. 5A

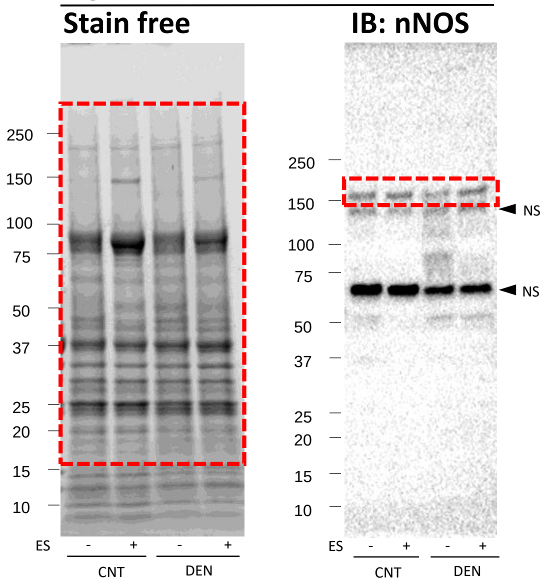

Fig. 5B

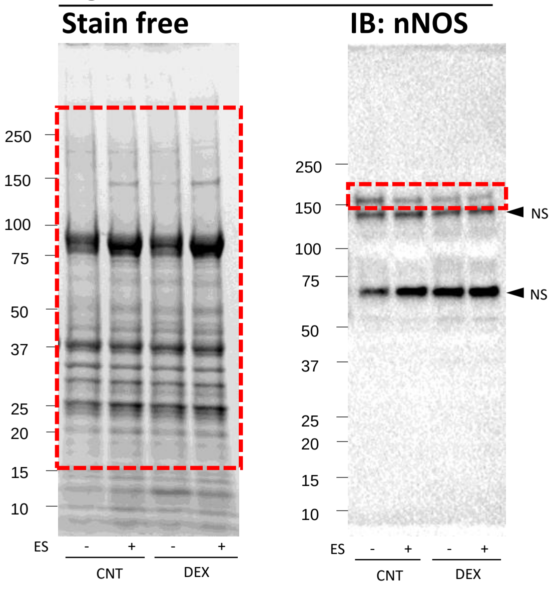

Fig. 5D

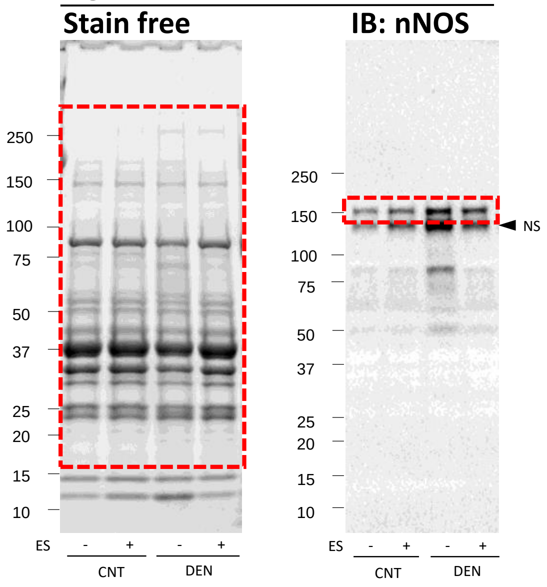

Fig. 5E

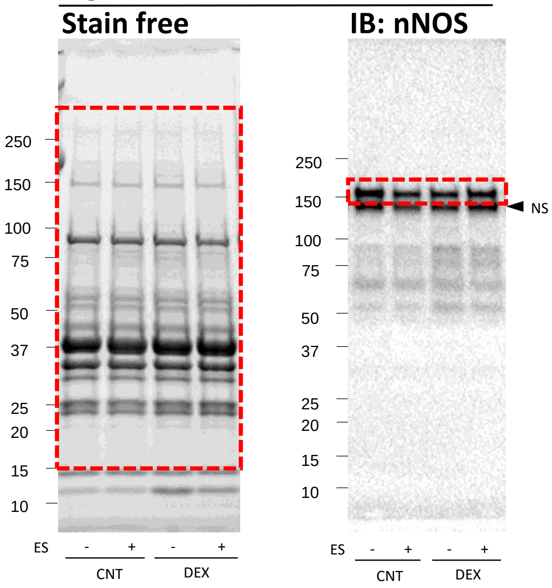

S1_raw_images. Original uncropped blots used for Fig. 5. Image was captured using a charge-coupled device camera attached to ChemiDOC MP (BioRad) and Image Lab Software was used for detection as well as densitometry. The part of the blot or gel shown in the manuscript is within the red box.
NS: non-specific binding.

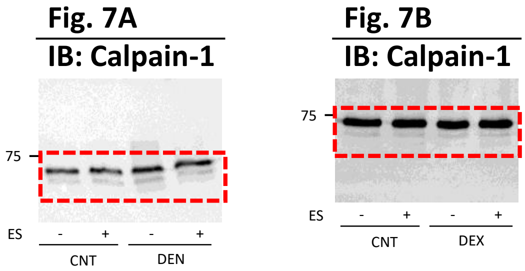

S1_raw_images. Original uncropped blots used for Fig. 7. Image was captured using a charge-coupled device camera attached to ChemiDOC MP (BioRad) and Image Lab Software was used for detection as well as densitometry. The part of the blot shown in the manuscript is within the red boxes
